# Supplementary material for: Explainable artificial intelligence toward usable and trustworthy computer-aided diagnosis of multiple sclerosis from Optical Coherence Tomography
Source: PLoS One. 2023 Aug 7;18(8):e0289495. doi: 10.1371/journal.pone.0289495 (PMC10406231; doi:10.1371/journal.pone.0289495)
Supplement: S1 File — (PDF) [file pone.0289495.s001.pdf]

Noname manuscript No.
(will be inserted by the editor)

Supplementary Material: Explainable artificial intelligence toward usable and trustworthy computer aided diagnosis of multiple sclerosis from optical coherence tomography

Monica Hernandez, Ubaldo Ramon-Julvez, Elisa Vilades, Beatriz Cordon, Elvira Mayordomo, Elena Garcia-Martin

Received: date / Accepted: date

Abstract This document accompanies our work *Explainable artificial intelligence toward usable and trustworthy computer aided diagnosis of multiple sclerosis from optical coherence tomography.* In it we show additional Tables and Figures of our experiments for a complementary understanding of our study.

List of Tables

1 Left eye sample (L). Performance results of gradient boosting (XGB), random forests (RF), explainable boosting machine (EBM), and EBM with interactions (EBM + i) on the Zones and the Posterior Pole (PPole) grid feature sets. The first row in each group shows the mean and the standard deviation of the corresponding metrics obtained in the ten-fold cross-validation. The second row in each group shows de max - min range obtained in the ten-fold cross-validation. Subscript Z indicates the Zones feature set. 3

2 Right eye sample (R). Performance results of gradient boosting (XGB), random forests (RF), explainable boosting machine (EBM), and EBM with interactions (EBM + i) on the Zones and the Posterior Pole (PPole) grid feature sets. The first row in each group shows the mean and the standard deviation of the corresponding metrics obtained in the ten-fold cross-validation. The second row in each group shows de max - min range obtained in the ten-fold cross-validation. Subscript Z indicates the Zones feature set. 4

M. Hernandez
University of Zaragoza
Tel.: +34-876-865544
Fax: +34-976-761914
E-mail: mhg@unizar.es

3 Random eye sample (rand). Performance results of gradient boosting (XGB), random forests (RF), explainable boosting machine (EBM), and EBM with interactions (EBM + i) on the Zones and the Posterior Pole (PPole) grid feature sets. The first row in each group shows the mean and the standard deviation of the corresponding metrics obtained in the ten-fold cross-validation. The second row in each group shows de max - min range obtained in the ten-fold cross-validation. Subscript Z indicates the Zones feature set. 5

4 Both eyes sample (LR). Performance results of gradient boosting (XGB), random forests (RF), explainable boosting machine (EBM), and EBM with interactions (EBM + i) on the Zones and the Posterior Pole (PPole) grid feature sets. The first row in each group shows the mean and the standard deviation of the corresponding metrics obtained in the ten-fold cross-validation. The second row in each group shows de max - min range obtained in the ten-fold cross-validation. Subscript Z indicates the Zones feature set. 6

List of Figures

1 Confusion matrices for the best and worst gradient boosting (XGB) and random forests (RF) models with the Posterior Pole grid feature set. 7

2 Confusion matrices for the best and worst Explainable Boosting Machine models. 7

3 Local SHAP results. Gradient boosting (XGB) worst-performing model. Left, Posterior Pole grid sample. Middle, grid of the local SHAP values. Right, waterfall plot for assesing feature contribution towards or against multiple sclerosis. 8

4 Local SHAP results. Gradient boosting (XGB) worst-performing model. Left, Posterior Pole grid sample. Middle, grid of the local SHAP values. Right, waterfall plot for assesing feature contribution towards or against multiple sclerosis. 9

5 Local SHAP results. Random forests (RF) worst-performing model. Left, Posterior Pole grid sample. Middle, grid of the local SHAP values. Right, waterfall plot for assesing feature contribution towards or against multiple sclerosis. . . 10

6 Local SHAP results. Random Forests (RF) worst-performing model. Left, Posterior Pole grid sample. Middle, grid of the local SHAP values. Right, waterfall plot for assesing feature contribution towards or against multiple sclerosis. 11

7 Local Explainable Boosting Machine (EBM) results. EBM worst-performing model. For each subject, left figure shows the PPole grid sample and right figure shows the feature contribution towards or against multiple sclerosis. . . 12

8 Local Explainable Boosting Machine (EBM) results. EBM worst-performing model. For each subject, left figure shows the PPole grid sample and right figure shows the feature contribution towards or against multiple sclerosis. . . 13

XGBClassifier	Accuracy (%)	Sensitivity (%)	Specificity (%)	F1-score (%)	AUC
GCL	75.22 ± 4.27	59.76 ± 9.68	81.89 ± 6.90	55.88 ± 9.75	0.71 ± 0.04
GCL	82.76 - 67.74	71.43 - 44.44	90.48 - 72.00	66.67 - 33.33	0.77 - 0.63
GCL_Z	79.36 ± 5.22	63.70 ± 11.03	86.17 ± 6.45	61.90 ± 11.75	0.75 ± 0.06
GCL_Z	86.67 - 71.43	85.71 - 45.45	95.00 - 72.00	75.00 - 33.33	0.85 - 0.68
RF	Accuracy (%)	Sensitivity (%)	Specificity (%)	F1-score (%)	AUC
GCL	75.58 ± 5.20	50.90 ± 12.67	85.32 ± 6.19	52.41 ± 14.77	0.68 ± 0.08
GCL	83.87 - 67.86	66.67 - 33.33	95.00 - 72.00	73.68 - 18.18	0.79 - 0.53
GCL_Z	76.59 ± 5.36	63.20 ± 10.71	82.57 ± 8.40	58.74 ± 12.00	0.73 ± 0.05
GCL_Z	81.48 - 64.29	85.71 - 50.00	95.00 - 64.00	70.59 - 28.57	0.83 - 0.65
EBM	Accuracy (%)	Sensitivity (%)	Specificity (%)	F1-score (%)	AUC
GCL	74.86 ± 7.52	52.01 ± 21.35	83.05 ± 8.13	53.08 ± 20.07	0.68 ± 0.14
GCL	82.76 - 57.14	83.33 - 0.00	95.00 - 64.00	66.67 - 0.00	0.83 - 0.32
GCL_Z	75.02 ± 8.95	59.52 ± 13.45	81.65 ± 10.68	56.11 ± 15.31	0.71 ± 0.09
GCL_Z	85.19 - 60.71	85.71 - 40.00	95.00 - 60.00	75.00 - 26.67	0.85 - 0.60
EBM + i	Accuracy (%)	Sensitivity (%)	Specificity (%)	F1-score (%)	AUC
GCL	75.53 ± 6.58	45.12 ± 18.63	86.76 ± 7.82	50.07 ± 19.91	0.66 ± 0.12
GCL	86.21 - 64.29	66.67 - 0.00	100.00 - 72.00	70.59 - 0.00	0.79 - 0.36
GCL_Z	78.35 ± 6.83	64.71 ± 19.20	85.43 ± 8.70	60.71 ± 13.06	0.75 ± 0.08
GCL_Z	90.00 - 67.86	100.00 - 44.44	95.00 - 64.00	80.00 - 40.00	0.89 - 0.65
XGBClassifier	Accuracy (%)	Sensitivity (%)	Specificity (%)	F1-score (%)	AUC
RNFL	67.91 ± 5.46	36.35 ± 26.09	82.07 ± 7.43	34.42 ± 14.02	0.59 ± 0.12
RNFL	79.31 - 63.33	100.00 - 10.00	90.00 - 71.43	62.50 - 15.38	0.86 - 0.48
$RNFL_Z$	60.78 ± 10.04	38.05 ± 18.41	70.11 ± 9.21	33.57 ± 14.86	0.54 ± 0.12
$RNFL_Z$	75.00 - 40.00	66.67 - 11.11	85.00 - 52.38	52.63 - 10.00	0.71 - 0.32
RF	Accuracy (%)	Sensitivity (%)	Specificity (%)	F1-score (%)	AUC
RNFL	69.89 ± 4.54	29.51 ± 12.88	85.62 ± 4.97	33.35 ± 13.55	0.58 ± 0.06
RNFL	75.86 - 61.29	55.56 - 9.09	95.00 - 80.00	58.82 - 14.29	0.70 - 0.50
$RNFL_Z$	62.88 ± 9.70	27.33 ± 12.98	75.57 ± 11.21	29.30 ± 13.73	0.51 ± 0.09
$RNFL_Z$	76.67 - 43.33	44.44 - 0.00	91.30 - 57.14	44.44 - 0.00	0.60 - 0.34
EBM	Accuracy (%)	Sensitivity (%)	Specificity (%)	F1-score (%)	AUC
RNFL	66.53 ± 7.33	27.16 ± 16.98	82.11 ± 7.69	29.47 ± 17.78	0.55 ± 0.10
RNFL	79.31 - 56.67	55.56 - 0.00	95.00 - 72.00	62.50 - 0.00	0.73 - 0.37
$RNFL_Z$	56.84 ± 10.91	38.70 ± 13.20	63.25 ± 11.37	32.64 ± 12.73	0.51 ± 0.10
$RNFL_Z$	74.07 - 40.00	63.64 - 22.22	80.00 - 45.00	56.00 - 18.18	0.69 - 0.35
EBM + i	Accuracy (%)	Sensitivity (%)	Specificity (%)	F1-score (%)	AUC
RNFL	67.22 ± 6.46	32.50 ± 13.68	81.03 ± 8.33	33.68 ± 14.95	0.57 ± 0.07
RNFL	75.86 - 56.67	50.00 - 9.09	90.00 - 66.67	53.33 - 14.29	0.67 - 0.48
$RNFL_Z$	58.76 ± 6.67	38.56 ± 13.22	66.47 ± 8.48	33.22 ± 12.40	0.53 ± 0.08
$RNFL_Z$	70.37 - 43.33	60.00 - 20.00	80.00 - 52.38	50.00 - 14.29	0.61 - 0.37

Table 1 Left eye sample (L). Performance results of gradient boosting (XGB), random forests (RF), explainable boosting machine (EBM), and EBM with interactions (EBM + i) on the Zones and the Posterior Pole (PPole) grid feature sets. The first row in each group shows the mean and the standard deviation of the corresponding metrics obtained in the ten-fold cross-validation. The second row in each group shows de max - min range obtained in the ten-fold cross-validation. Subscript Z indicates the Zones feature set.

XGBClassifier	Accuracy (%)	Sensitivity (%)	Specificity (%)	F1-score (%)	AUC
GCL	81.70 ± 7.34	66.11 ± 18.02	89.18 ± 3.83	67.94 ± 12.57	0.78 ± 0.10
GCL	90.32 - 66.67	100.00 - 30.00	95.65 - 85.00	82.35 - 37.50	0.93 - 0.57
GCL_Z	78.39 ± 5.13	68.03 ± 14.81	83.21 ± 3.11	64.54 ± 10.21	0.76 ± 0.08
GCL_Z	87.10 - 70.00	87.50 - 40.00	90.00 - 80.00	81.82 - 47.06	0.86 - 0.62
RF	Accuracy (%)	Sensitivity (%)	Specificity (%)	F1-score (%)	AUC
GCL	82.66 ± 7.31	64.80 ± 14.60	90.75 ± 4.75	68.46 ± 13.81	0.78 ± 0.09
GCL	90.32 - 66.67	81.82 - 30.00	100.00 - 85.00	85.71 - 37.50	0.88 - 0.57
GCL_Z	80.66 ± 5.98	68.20 ± 15.41	86.44 ± 6.18	67.16 ± 11.81	0.77 ± 0.07
GCL_Z	90.32 - 70.00	87.50 - 30.00	95.65 - 76.19	81.82 - 40.00	0.86 - 0.60
EBM	Accuracy (%)	Sensitivity (%)	Specificity (%)	F1-score (%)	AUC
GCL	77.35 ± 6.82	54.52 ± 19.74	87.65 ± 7.06	57.10 ± 17.01	0.71 ± 0.10
GCL	90.32 - 66.67	81.82 - 20.00	100.00 - 78.26	85.71 - 28.57	0.88 - 0.55
GCL_Z	77.31 ± 4.47	65.10 ± 14.30	82.67 ± 7.96	62.57 ± 9.56	0.74 ± 0.06
GCL_Z	84.38 - 70.00	87.50 - 42.86	95.00 - 66.67	75.00 - 40.00	0.81 - 0.61
EBM + i	Accuracy (%)	Sensitivity (%)	Specificity (%)	F1-score (%)	AUC
GCL	79.65 ± 5.96	60.92 ± 16.15	88.46 ± 4.93	63.10 ± 13.65	0.75 ± 0.08
GCL	87.10 - 66.67	75.00 - 20.00	95.00 - 80.95	80.00 - 28.57	0.84 - 0.55
GCL_Z	79.37 ± 5.55	65.71 ± 7.94	85.45 ± 5.53	65.61 ± 8.28	0.76 ± 0.06
GCL_Z	87.10 - 72.73	75.00 - 50.00	95.00 - 78.26	77.78 - 50.00	0.83 - 0.67
XGBClassifier	Accuracy (%)	Sensitivity (%)	Specificity (%)	F1-score (%)	AUC
RNFL	73.50 ± 6.49	51.49 ± 13.99	83.72 ± 5.93	53.19 ± 11.16	0.68 ± 0.07
RNFL	87.10 - 63.64	75.00 - 30.77	91.30 - 75.00	75.00 - 40.00	0.83 - 0.58
$RNFL_Z$	67.21 ± 4.16	46.55 ± 19.16	77.32 ± 10.00	44.18 ± 7.31	0.62 ± 0.06
$RNFL_Z$	74.19 - 62.07	85.71 - 22.22	95.00 - 64.00	54.55 - 33.33	0.75 - 0.55
RF	Accuracy (%)	Sensitivity (%)	Specificity (%)	F1-score (%)	AUC
RNFL	73.41 ± 5.91	47.42 ± 18.31	85.34 ± 7.25	50.12 ± 14.15	0.66 ± 0.09
RNFL	83.87 - 65.52	75.00 - 22.22	95.00 - 76.00	76.19 - 28.57	0.81 - 0.54
$RNFL_Z$	69.23 ± 7.45	46.96 ± 16.02	79.29 ± 10.99	46.97 ± 12.42	0.63 ± 0.08
$RNFL_Z$	77.42 - 56.67	71.43 - 22.22	100.00 - 64.00	63.16 - 25.00	0.72 - 0.49
EBM	Accuracy (%)	Sensitivity (%)	Specificity (%)	F1-score (%)	AUC
RNFL	70.86 ± 8.29	47.04 ± 16.25	81.61 ± 10.07	48.20 ± 14.88	0.64 ± 0.09
RNFL	83.87 - 60.61	72.73 - 20.00	95.00 - 66.67	76.19 - 30.77	0.81 - 0.55
$RNFL_Z$	66.47 ± 8.76	57.85 ± 20.28	70.42 ± 12.50	50.08 ± 13.24	0.64 ± 0.10
$RNFL_Z$	87.10 - 56.67	88.89 - 33.33	91.30 - 55.00	75.00 - 35.29	0.83 - 0.55
EBM + i	Accuracy (%)	Sensitivity (%)	Specificity (%)	F1-score (%)	AUC
RNFL	69.15 ± 2.80	47.35 ± 15.39	79.26 ± 8.47	46.66 ± 9.21	0.63 ± 0.05
RNFL	74.19 - 63.33	71.43 - 28.57	90.00 - 66.67	63.64 - 28.57	0.72 - 0.53
$RNFL_Z$	69.09 ± 8.76	53.49 ± 24.85	76.25 ± 11.30	49.17 ± 16.94	0.65 ± 0.12
$RNFL_Z$	87.10 - 55.17	88.89 - 22.22	91.30 - 60.00	75.00 - 23.53	0.83 - 0.46

Table 2 Right eye sample (R). Performance results of gradient boosting (XGB), random forests (RF), explainable boosting machine (EBM), and EBM with interactions (EBM + i) on the Zones and the Posterior Pole (PPole) grid feature sets. The first row in each group shows the mean and the standard deviation of the corresponding metrics obtained in the ten-fold cross-validation. The second row in each group shows de max - min range obtained in the ten-fold cross-validation. Subscript Z indicates the Zones feature set.

XGBClassifier	Accuracy (%)	Sensitivity (%)	Specificity (%)	F1-score (%)	AUC
GCL	78.58 ± 4.98	67.06 ± 16.82	84.22 ± 7.31	63.11 ± 8.29	0.76 ± 0.07
GCL	87.10 - 73.33	100.00 - 40.00	95.00 - 73.91	81.82 - 53.33	0.92 - 0.67
GCL_Z	81.56 ± 7.94	75.22 ± 13.14	84.87 ± 9.59	69.36 ± 13.26	0.80 ± 0.08
GCL_Z	93.55 - 68.97	90.91 - 55.56	100.00 - 75.00	90.91 - 52.63	0.93 - 0.65
RF	Accuracy (%)	Sensitivity (%)	Specificity (%)	F1-score (%)	AUC
GCL	80.26 ± 5.74	60.31 ± 22.18	89.39 ± 4.37	61.49 ± 14.05	0.75 ± 0.10
GCL	87.10 - 70.00	100.00 - 20.00	95.24 - 84.00	81.82 - 30.77	0.92 - 0.57
GCL_Z	79.61 ± 9.41	67.10 ± 19.37	85.23 ± 7.19	64.11 ± 17.44	0.76 ± 0.11
GCL_Z	92.86 - 63.33	90.00 - 30.00	95.00 - 72.00	87.50 - 35.29	0.91 - 0.55
EBM	Accuracy (%)	Sensitivity (%)	Specificity (%)	F1-score (%)	AUC
GCL	79.63 ± 3.44	58.89 ± 17.27	89.03 ± 4.95	60.88 ± 9.04	0.74 ± 0.07
GCL	83.87 - 73.33	100.00 - 30.00	95.00 - 80.00	73.68 - 42.86	0.90 - 0.62
GCL_Z	76.94 ± 6.73	60.01 ± 17.37	84.26 ± 8.88	58.81 ± 12.50	0.72 ± 0.09
GCL_Z	85.71 - 66.67	85.71 - 33.33	100.00 - 70.00	76.19 - 37.50	0.82 - 0.57
EBM + i	Accuracy (%)	Sensitivity (%)	Specificity (%)	F1-score (%)	AUC
GCL	80.65 ± 5.60	63.30 ± 21.28	88.31 ± 5.44	63.36 ± 13.51	0.76 ± 0.09
GCL	90.00 - 70.00	100.00 - 20.00	95.24 - 80.95	78.26 - 30.77	0.94 - 0.57
GCL_Z	77.61 ± 7.24	66.84 ± 15.15	82.20 ± 7.34	62.26 ± 12.32	0.75 ± 0.09
GCL_Z	90.32 - 66.67	85.71 - 44.44	95.00 - 75.00	85.71 - 42.86	0.88 - 0.62
XGBClassifier	Accuracy (%)	Sensitivity (%)	Specificity (%)	F1-score (%)	AUC
RNFL	69.88 ± 6.97	47.82 ± 22.30	78.97 ± 8.81	45.35 ± 15.63	0.63 ± 0.10
RNFL	83.33 - 61.29	80.00 - 16.67	95.00 - 65.22	69.57 - 18.18	0.82 - 0.50
$RNFL_Z$	69.26 ± 8.19	52.40 ± 18.28	75.99 ± 6.65	48.29 ± 14.74	0.64 ± 0.11
$RNFL_Z$	78.57 - 50.00	83.33 - 28.57	82.61 - 60.00	66.67 - 28.57	0.79 - 0.45
RF	Accuracy (%)	Sensitivity (%)	Specificity (%)	F1-score (%)	AUC
RNFL	72.16 ± 6.49	44.40 ± 21.88	84.41 ± 7.73	44.98 ± 17.23	0.64 ± 0.10
RNFL	83.87 - 64.52	80.00 - 11.11	95.24 - 72.00	76.19 - 16.67	0.81 - 0.51
$RNFL_Z$	71.94 ± 5.26	44.48 ± 12.30	83.17 ± 4.66	46.67 ± 10.16	0.64 ± 0.06
$RNFL_Z$	79.31 - 63.33	66.67 - 30.00	90.00 - 76.19	63.64 - 33.33	0.75 - 0.55
EBM	Accuracy (%)	Sensitivity (%)	Specificity (%)	F1-score (%)	AUC
RNFL	69.71 ± 7.38	45.21 ± 18.66	80.26 ± 6.71	44.39 ± 12.91	0.63 ± 0.09
RNFL	82.76 - 61.29	75.00 - 20.00	95.00 - 70.00	66.67 - 26.67	0.78 - 0.51
$RNFL_Z$	62.25 ± 6.44	38.10 ± 15.40	71.70 ± 10.11	35.08 ± 11.66	0.55 ± 0.06
$RNFL_Z$	72.41 - 48.39	63.64 - 11.11	85.71 - 57.14	53.85 - 15.38	0.64 - 0.44
EBM + i	Accuracy (%)	Sensitivity (%)	Specificity (%)	F1-score (%)	AUC
RNFL	68.98 ± 5.97	43.17 ± 23.58	80.94 ± 6.10	41.25 ± 14.38	0.62 ± 0.10
RNFL	76.67 - 60.00	80.00 - 10.00	90.00 - 75.00	63.16 - 15.38	0.78 - 0.49
$RNFL_Z$	63.31 ± 7.63	34.36 ± 13.63	75.18 ± 7.98	34.22 ± 11.27	0.55 ± 0.09
$RNFL_Z$	73.33 - 50.00	60.00 - 14.29	85.71 - 65.00	47.06 - 12.50	0.68 - 0.40

Table 3 Random eye sample (rand). Performance results of gradient boosting (XGB), random forests (RF), explainable boosting machine (EBM), and EBM with interactions (EBM + i) on the Zones and the Posterior Pole (PPole) grid feature sets. The first row in each group shows the mean and the standard deviation of the corresponding metrics obtained in the ten-fold cross-validation. The second row in each group shows de max - min range obtained in the ten-fold cross-validation. Subscript Z indicates the Zones feature set.

XGBClassifier	Accuracy (%)	Sensitivity (%)	Specificity (%)	F1-score (%)	AUC
GCL	79.50 ± 3.93	64.77 ± 12.51	86.14 ± 4.46	63.73 ± 8.42	0.75 ± 0.06
GCL	86.67 - 73.33	85.71 - 50.00	90.48 - 78.26	80.00 - 51.85	0.85 - 0.69
GCL_Z	81.17 ± 4.01	73.02 ± 10.64	84.50 ± 4.79	68.46 ± 7.88	0.79 ± 0.05
GCL_Z	88.33 - 75.00	85.00 - 55.00	93.48 - 78.00	82.93 - 55.17	0.88 - 0.70
RF	Accuracy (%)	Sensitivity (%)	Specificity (%)	F1-score (%)	AUC
GCL	81.33 ± 4.83	65.46 ± 14.51	88.57 ± 5.13	65.74 ± 11.59	0.77 ± 0.07
GCL	88.33 - 71.67	80.00 - 30.00	95.00 - 78.00	82.05 - 41.38	0.86 - 0.61
GCL_Z	79.83 ± 5.00	66.39 ± 10.33	85.78 ± 5.34	65.07 ± 8.66	0.76 ± 0.06
GCL_Z	86.67 - 71.67	78.57 - 45.00	92.50 - 77.50	78.95 - 50.00	0.84 - 0.67
EBM	Accuracy (%)	Sensitivity (%)	Specificity (%)	F1-score (%)	AUC
GCL	77.67 ± 3.70	59.74 ± 9.97	85.43 ± 4.40	59.95 ± 8.46	0.73 ± 0.05
GCL	83.33 - 71.67	75.00 - 45.00	92.50 - 80.00	75.00 - 46.15	0.81 - 0.65
GCL_Z	79.00 ± 4.17	68.78 ± 11.18	83.58 ± 4.91	64.59 ± 9.31	0.76 ± 0.05
GCL_Z	88.33 - 75.00	90.00 - 50.00	87.50 - 76.00	83.72 - 48.28	0.89 - 0.69
EBM + i	Accuracy (%)	Sensitivity (%)	Specificity (%)	F1-score (%)	AUC
GCL	79.17 ± 4.39	59.58 ± 12.76	87.78 ± 4.43	61.26 ± 10.27	0.74 ± 0.06
GCL	86.67 - 71.67	71.43 - 30.00	95.00 - 82.00	77.78 - 41.38	0.82 - 0.61
GCL_Z	80.17 ± 6.16	70.23 ± 13.49	84.48 ± 5.95	66.29 ± 11.98	0.77 ± 0.08
GCL_Z	93.33 - 73.33	95.00 - 45.00	92.50 - 74.00	90.48 - 46.67	0.94 - 0.66
XGBClassifier	Accuracy (%)	Sensitivity (%)	Specificity (%)	F1-score (%)	AUC
RNFL	72.17 ± 4.01	52.44 ± 9.78	80.76 ± 6.28	51.45 ± 7.55	0.67 ± 0.05
RNFL	78.33 - 66.67	72.22 - 35.00	90.00 - 71.74	62.86 - 41.18	0.73 - 0.60
$RNFL_Z$	65.67 ± 6.49	36.17 ± 15.80	78.03 ± 7.10	36.71 ± 12.33	0.57 ± 0.08
$RNFL_Z$	75.00 - 56.67	60.00 - 14.29	89.13 - 69.05	61.54 - 13.33	0.71 - 0.42
RF	Accuracy (%)	Sensitivity (%)	Specificity (%)	F1-score (%)	AUC
RNFL	73.50 ± 2.66	49.71 ± 9.79	83.98 ± 6.28	51.15 ± 7.51	0.67 ± 0.04
RNFL	78.33 - 70.00	65.00 - 35.00	92.50 - 74.00	66.67 - 41.38	0.75 - 0.61
$RNFL_Z$	67.33 ± 5.94	40.65 ± 14.18	78.84 ± 6.20	40.95 ± 10.56	0.60 ± 0.08
$RNFL_Z$	75.00 - 55.00	70.00 - 22.22	86.96 - 69.05	57.89 - 22.86	0.71 - 0.46
EBM	Accuracy (%)	Sensitivity (%)	Specificity (%)	F1-score (%)	AUC
RNFL	71.00 ± 6.05	46.39 ± 12.26	81.80 ± 9.06	47.21 ± 10.13	0.64 ± 0.06
RNFL	81.67 - 63.33	65.00 - 25.00	92.50 - 67.39	70.27 - 35.71	0.77 - 0.56
$RNFL_Z$	60.50 ± 8.32	53.71 ± 8.33	63.01 ± 11.17	44.17 ± 10.50	0.58 ± 0.07
$RNFL_Z$	71.67 - 45.00	70.00 - 40.00	76.09 - 40.48	56.00 - 21.62	0.67 - 0.47
EBM + i	Accuracy (%)	Sensitivity (%)	Specificity (%)	F1-score (%)	AUC
RNFL	70.17 ± 4.48	47.56 ± 13.71	80.16 ± 7.64	46.70 ± 9.96	0.64 ± 0.06
RNFL	76.67 - 61.67	66.67 - 25.00	92.50 - 71.43	63.16 - 35.71	0.73 - 0.55
$RNFL_Z$	65.00 ± 5.15	42.60 ± 16.27	74.97 ± 7.36	40.11 ± 10.04	0.59 ± 0.08
$RNFL_Z$	75.00 - 58.33	80.00 - 28.57	82.50 - 64.29	61.54 - 24.24	0.73 - 0.48

Table 4 Both eyes sample (LR). Performance results of gradient boosting (XGB), random forests (RF), explainable boosting machine (EBM), and EBM with interactions (EBM + i) on the Zones and the Posterior Pole (PPole) grid feature sets. The first row in each group shows the mean and the standard deviation of the corresponding metrics obtained in the ten-fold cross-validation. The second row in each group shows de max - min range obtained in the ten-fold cross-validation. Subscript Z indicates the Zones feature set.

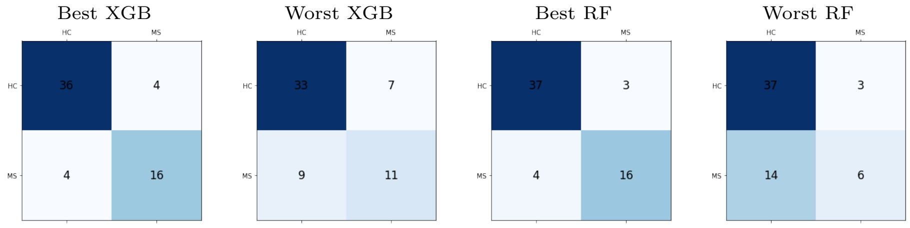

Fig. 1 Confusion matrices for the best and worst gradient boosting (XGB) and random forests (RF) models with the Posterior Pole grid feature set.

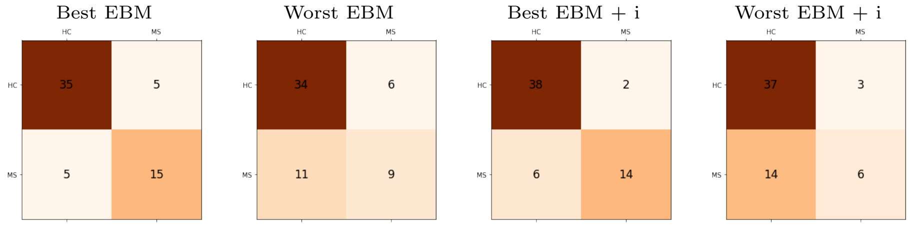

Fig. 2 Confusion matrices for the best and worst Explainable Boosting Machine models.

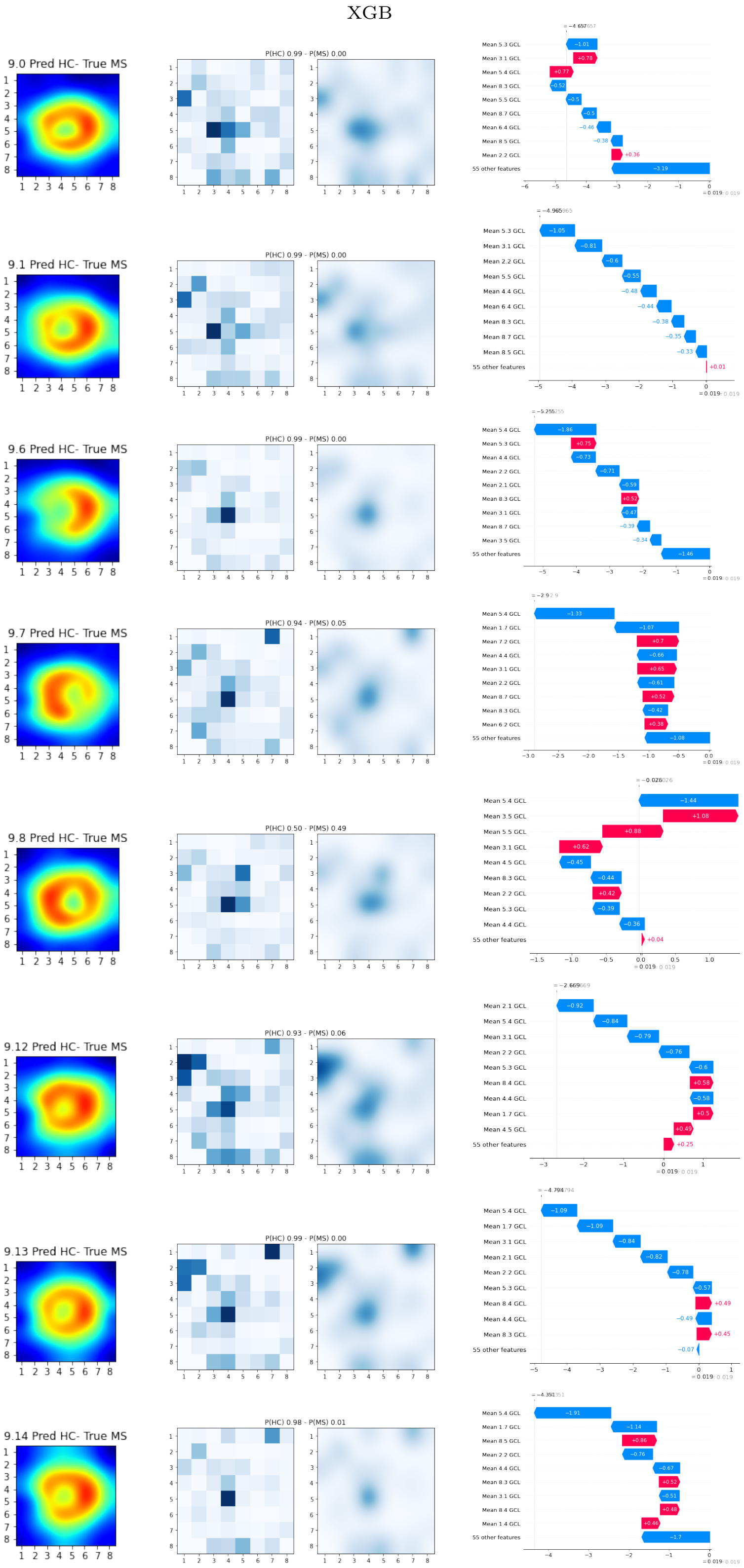

Fig. 3 Local SHAP results. Gradient boosting (XGB) worst-performing model. Left, Posterior Pole grid sample. Middle, grid of the local SHAP values. Right, waterfall plot for assesing feature contribution towards or against multiple sclerosis.

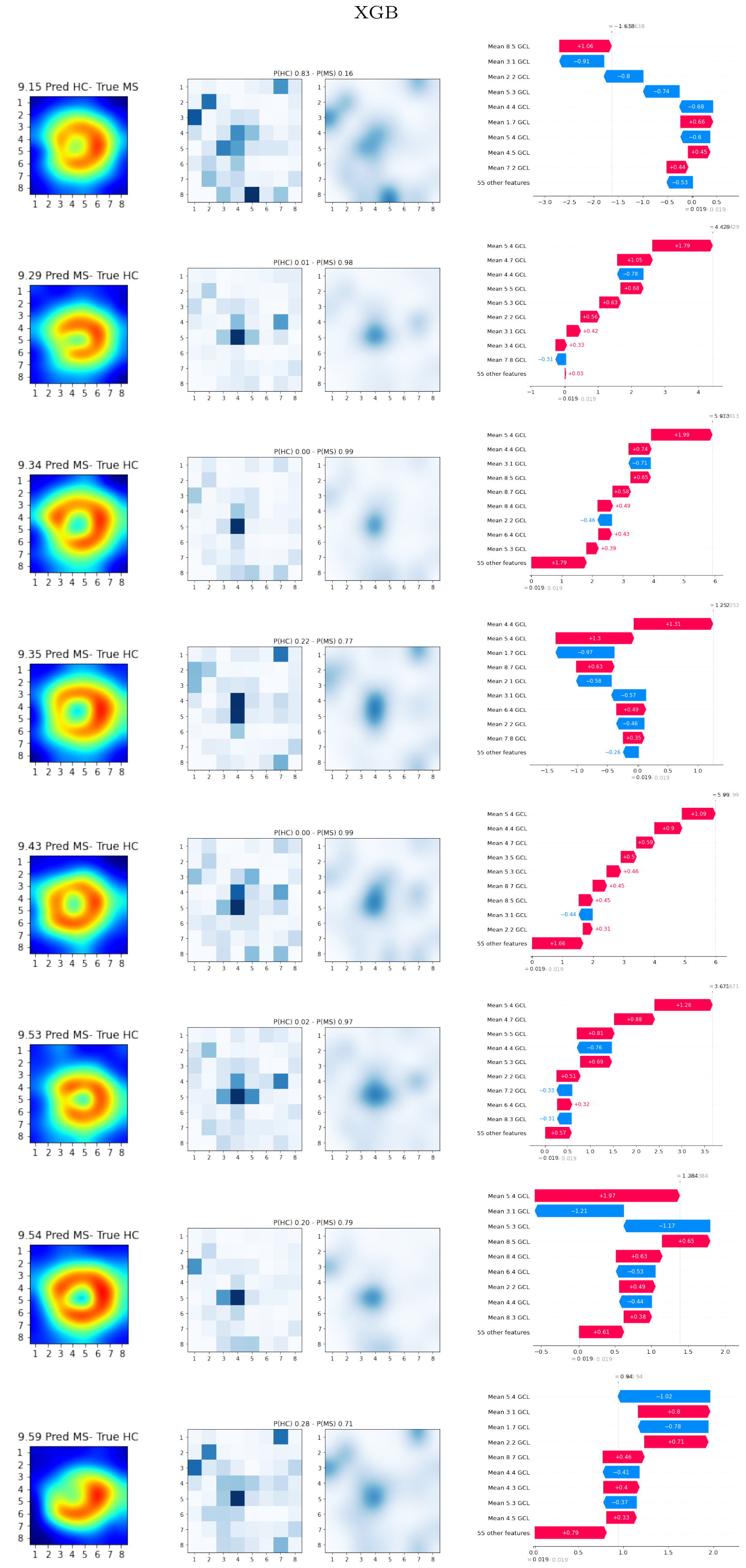

Fig. 4 Local SHAP results. Gradient boosting (XGB) worst-performing model. Left, Posterior Pole grid sample. Middle, grid of the local SHAP values. Right, waterfall plot for assesing feature contribution towards or against multiple sclerosis.

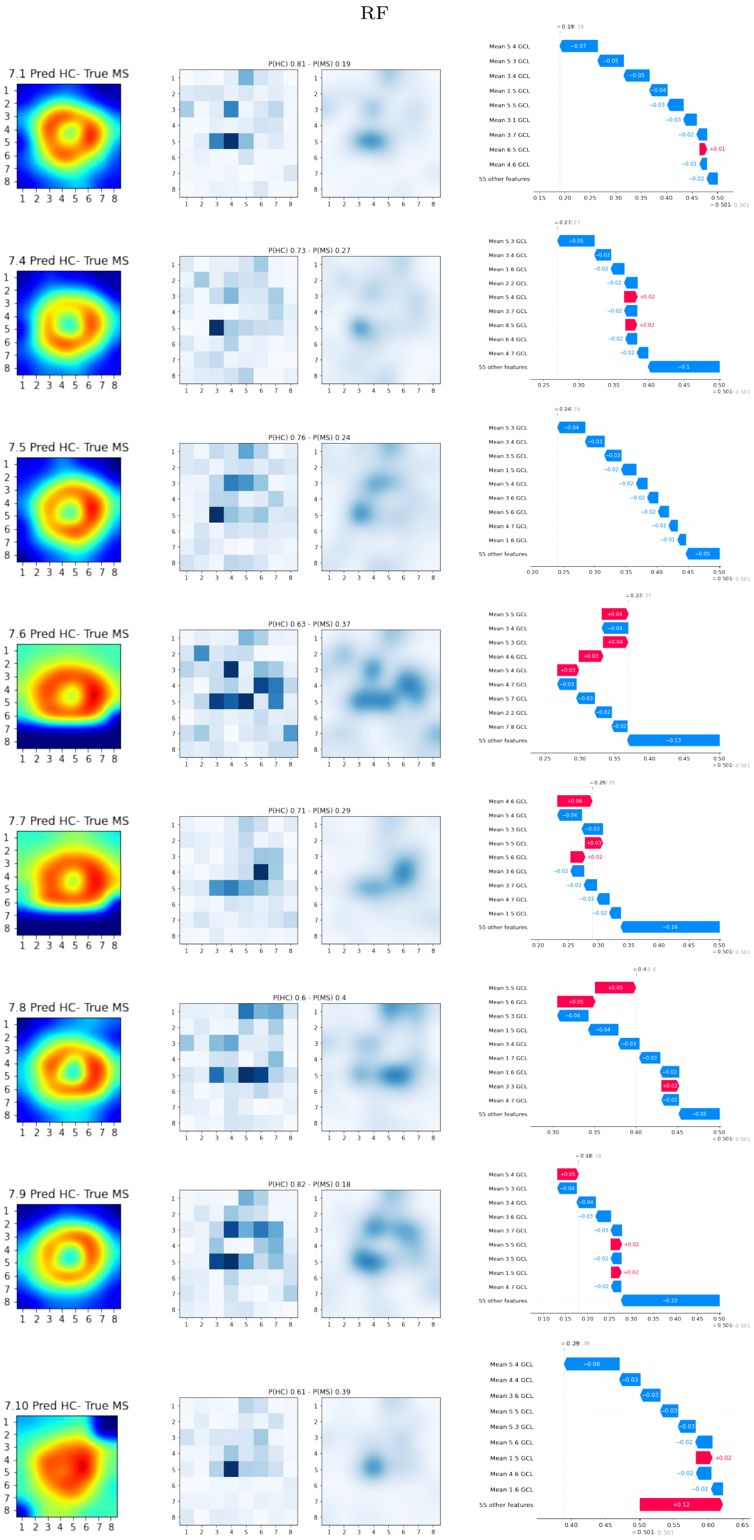

Fig. 5 Local SHAP results. Random forests (RF) worst-performing model. Left, Posterior Pole grid sample. Middle, grid of the local SHAP values. Right, waterfall plot for assesing feature contribution towards or against multiple sclerosis.

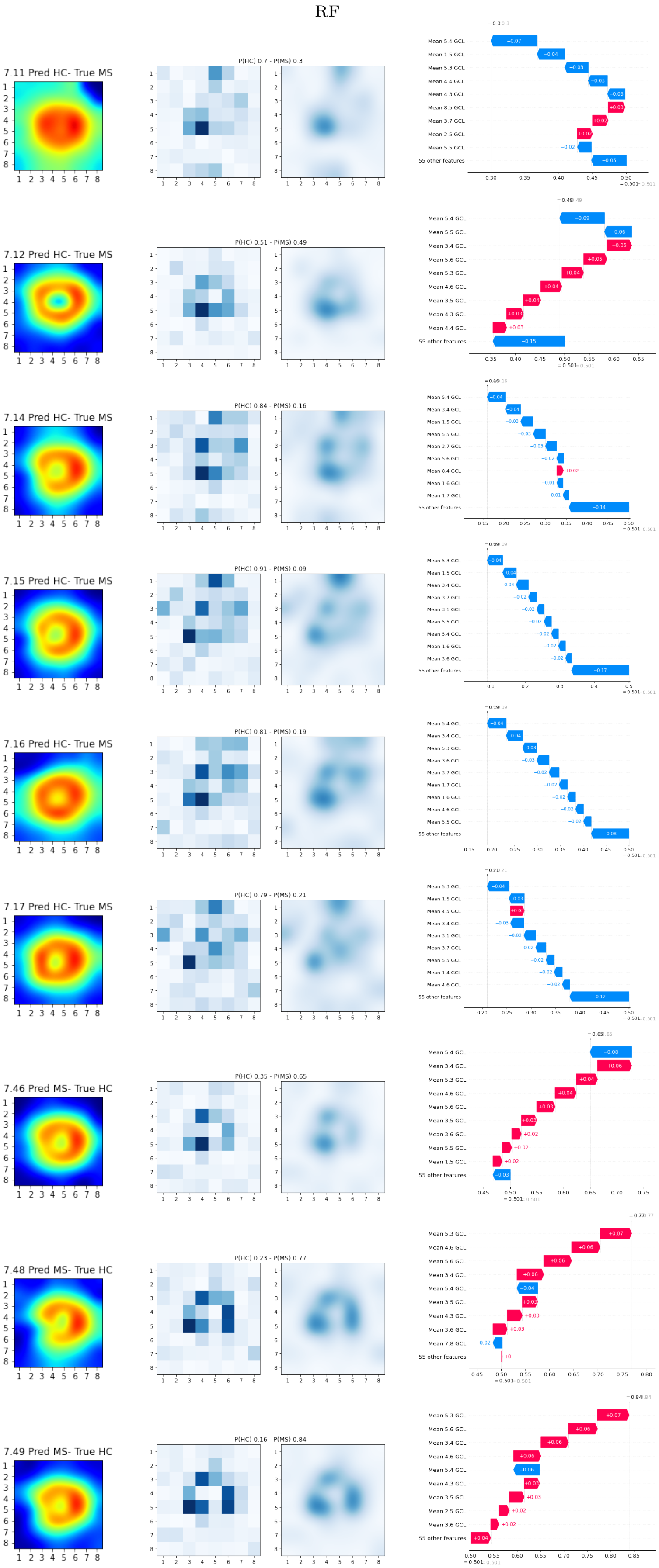

Fig. 6 Local SHAP results. Random Forests (RF) worst-performing model. Left, Posterior Pole grid sample. Middle, grid of the local SHAP values. Right, waterfall plot for assesing feature contribution towards or against multiple sclerosis.

EBM

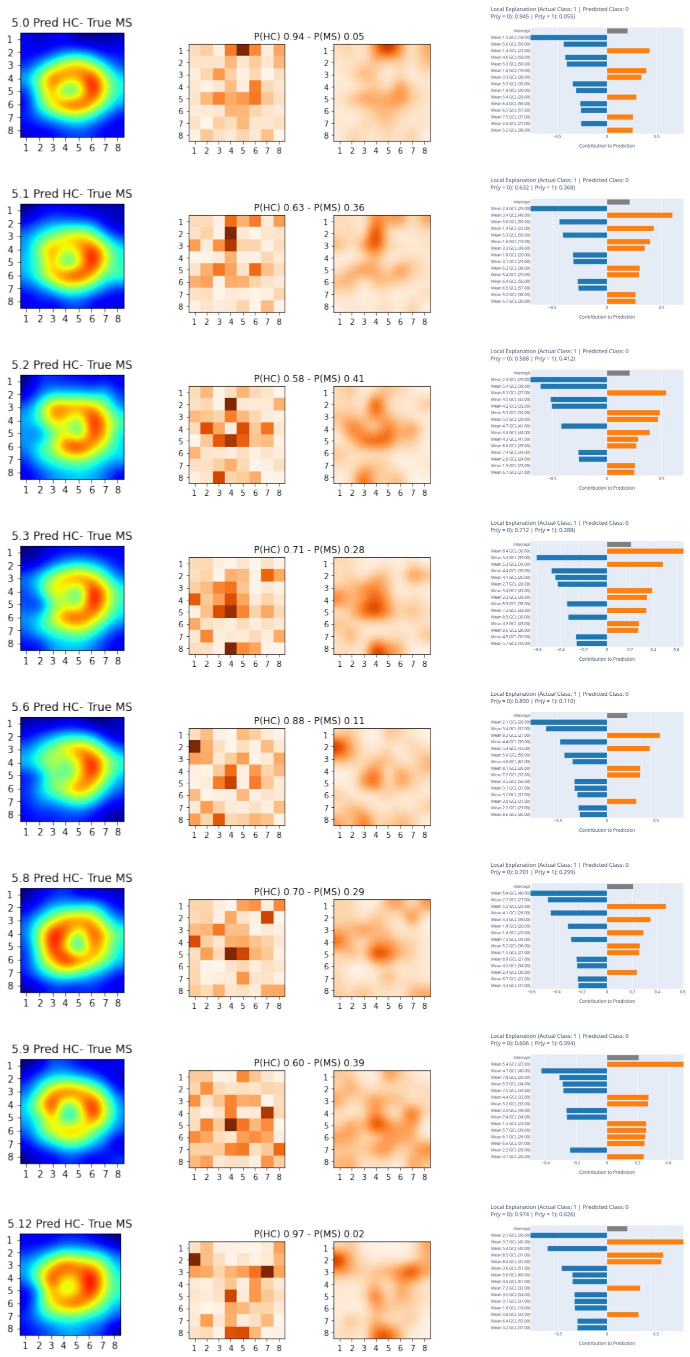

Fig. 7 Local Explainable Boosting Machine (EBM) results. EBM worst-performing model. For each subject, left figure shows the PPole grid sample and right figure shows the feature contribution towards or against multiple sclerosis.

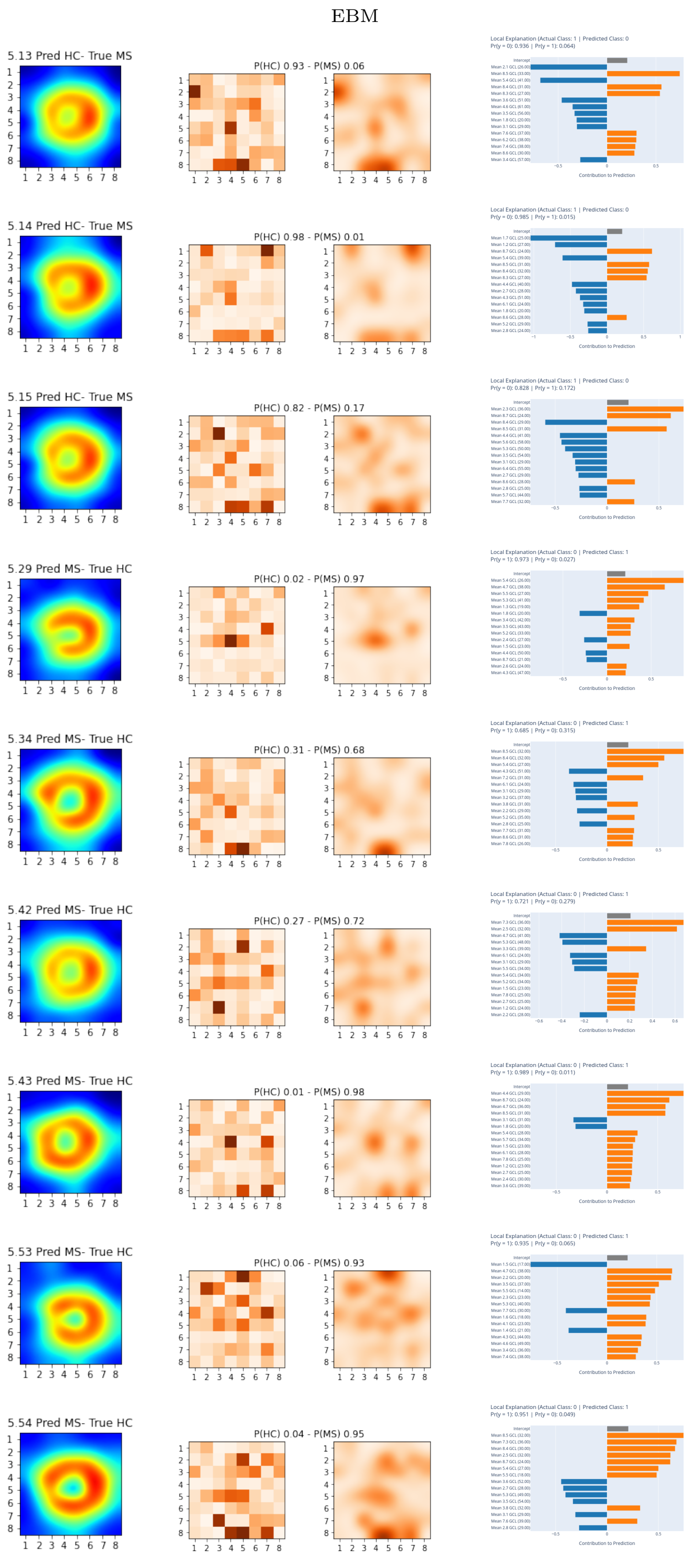

Fig. 8 Local Explainable Boosting Machine (EBM) results. EBM worst-performing model. For each subject, left figure shows the PPole grid sample and right figure shows the feature contribution towards or against multiple sclerosis.
